# Transgenic barley over-expressing *Aspergillus niger* phytase phyA in field trials

**DOI:** 10.1080/21645698.2025.2559488

**Published:** 2025-09-15

**Authors:** T Vlčko, V Psota, R Koprna, W Harwood, L Ohnoutková

**Affiliations:** a Laboratory of Growth Regulators, Institute of Experimental Botany, Czech Academy of Sciences, Olomouc, Czech Republic; b Research Institute of Brewing and Malting, Brno, Czech Republic; c Department of Chemical Biology, Faculty of Science, Palacký University, Olomouc, Czech Republic; d Department of Crop Genetics, John Innes Centre, Norwich Research Park, Norwich, UK; e Laboratory of Growth Regulators, Faculty of Science, Palacký University, Olomouc, Czech Republic

**Keywords:** Transgenic barley, phytase, androgenesis, hybridization, field trials

## Abstract

Phytic acid is the main storage of phosphate in grains of staple crops. As phytic acid is hardly digestible for non-ruminants microbial phytases are used to supplement animal feed to enhance phosphate digestibility. A fungal phytase gene was introduced into barley with the aim of enhancing phosphate digestibility. Transgenic homozygous barley over-expressing fungal phytase phyA showed a 3.3fold increase in mature grain phytase activity. Field trials at two locations in the Czech Republic were conducted in a five-year experiment to test transgene stability and activity under field conditions. Increased phytase activity gradually decreased over the generations showing the most significant drop in the initial years of field trials. Molecular analysis revealed methylation in the coding sequence of the *phyA* transgene, suggesting transcription gene silencing. On the other hand, herbicide resistance used for selection of transgenic plants was functional over all generations. The feasibility of crossing the transgene into the feeding cultivar Azit was demonstrated with subsequent stabilization of hybrid progeny through androgenesis. Our results indicate that the Azit genetic background tended to reduce phytase activity in mature grains of hybrids. Grain-specific over-expression of fungal phytase driven by an amylase promoter improved phosphate levels during germination. Unfortunately, a malting experiment revealed that phytase over-expression did not significantly improve malting parameters. In fact, the higher nitrogen content in unmalted grain negatively affected the quality of the malt produced from them.

## Introduction

Phosphorus is an essential nutrient in plant metabolism, an integral part of nucleic acids, phospholipid membranes, ATP, etc. In crop plants like barley, wheat, and maize, the grain’s phosphate content is an important nutritional parameter. To maintain high annual yields, large quantities of rock phosphate are utilized as fertilizer each year. However, reserves of rock phosphate are limited. Phosphate deposits are estimated to be depleted within 50 to 100 years. Moreover, the annual rate of consumption has increased fivefold in the last fifty years and is expected to increase further.^1^ Phosphate is the only form of phosphorus that plants can absorb through their roots. Higher plants store phosphate primarily in the form of organic polyphosphates, specifically *myo*-Inositolhexakisphosphate (IP6), commonly known as phytic acid. IP6 can form insoluble phytate complexes by associating with metal cations including Zn^2+^, Ca^2+^, and Fe^2+^. The majority of the synthesized IP6 is stored in protein storage vacuoles (PSV) located within the cells of the aleurone layer. Phytate is further aggregated into phytate globoids within the PSV.^2,3^

Phosphate seed reserves are mobilized for the evolving plant metabolism during germination. IP6 is enzymatically dephosphorylated by phytases that hydrolyze IP6 to lower inositol phosphates. Phytases actively maintain the optimal level of phosphate, contribute significantly to the mobilization of phosphate during germination and belong to a group of purple acid phosphatases.^4^ Based on the detection of the same inositol phosphates in mature grains as well as in grains during germination, Hatzack et al.^5^ suggested that phytases are active at a basal level even in mature grains. During germination, HvPAPhy_b1 and b2 are being *de novo* synthesized. Those two phytases are mainly responsible for phytate degradation.^4^ The content of IP6 in grains drops by about 80% after ten days. Most of the phytate is reduced by the 8^th^ day of germination coinciding with the highest phytase activity levels during the previous days.^6,7^ To improve the availability of phosphorus and minerals, the enzyme phytase is added to the feed, which increases the nutritional value of the feed, especially for monogastric animals.

There are two primary strategies for directly enhancing the proportion of digestible phosphate in plants. As a majority of phosphate is stored as IP6 in mature grains, it intuitively suggests two possible biotechnological approaches: reducing IP6 content or enhancing the enzymatic machinery capable of IP6 hydrolysis. IP6 content can be reduced by disrupting the IP6 biosynthetic pathway through mutagenesis of genes coding enzymes responsible for *myo*-Inositol phosphate synthesis and sequential phosphorylation. Many such “low phytic acid” lines have been developed.^8–13^ Alternatively, the activity of phytases in mature grains can be modulated via promoter editing,^14^ cis-genesis,^15^ or heterologous gene expression, which was broadly adapted over the years. Microbial phytase genes have been successfully transformed into agronomically important crops such as wheat,^16,17^ soy,^18^ canola^19,20^ or rice.^21,22^ The expression of these transgenes is typically driven by tissue-specific promoters, enabling specific expression in the grains of the transgenic plants.

Barley grain is used not only for feeding but also for malt and beer production. Microbial phytase in mature grains can release more phosphate from IP6, increasing the nutritional value of feed. Moreover, in addition to phosphate, microelements bound in the phytate complex are also released with hydrolyzed IP6. Increasing the content of bioavailable microelements improves the quality of the feed and can also increase the efficiency of fermentation in beer production.^23^

While genetically modified crops have been extensively evaluated in field trials since the 1990s, there is limited information on the assessment of transgenic barley expressing heterologous phytase. This study examines the performance of transgenic barley under field conditions at two locations with differing climatic conditions and soil types. A field trial with transgenic barley was conducted over five consecutive years to evaluate transgene stability and activity across an extended period. We evaluated the impact of a seed-specific amylase promoter driving the expression of the *phyA* phytase gene on phytase activity in mature grains of transgenic barley plants and during grain germination. Additionally, the key malting properties of the transgenic lines and their crosses with a feed cultivar were described. This manuscript presents a compelling dataset with implications for crop biotechnology, agriculture, highlighting the differential regulation of the *phyA* transgene and the selection gene for herbicide resistance.

## Materials and Methods

### Plant Material and Plasmid Constructs

Golden Promise is a malting, two-row spring barley variety. It is a mutant of the Maythorpe variety, which was treated with gamma radiation in 1956. The variety Golden Promise was officially registered in the United Kingdom in 1966. The variety remains popular with some craft brewers. The cultivar is frequently utilized in barley genetic research due to its exceptional responsiveness in tissue culture, rendering it a preferred subject for barley transformation.^24–26^ Azit is a feed, two-row spring barley variety that was registered in 2008. This variety has medium-high plants, medium-large grains, with a medium-high to high proportion of grains above 2.5 mm. It has medium resistance to a complex of leaf spots, minor resistance to powdery mildew, and minor resistance to lodging.

Transgenic barley was prepared as part of the project “Phytates removal and increased lysine content in cereals through the insertion of the phyt and dapA genes” within the European Framework program INCO-COPERNICUS, EC., (ERBIC 15 CT 961,011 PL 967,086) 1997–2000. The spring barley cultivar Golden Promise was used as the host for genetic transformation via particle bombardment, following established protocols.^27^ Preparation of transgenic plants was described in a previous study.^28^ Briefly, immature embryos were transformed with two plasmid vectors pAL70^29^ and pAMFIT (kindly provided by prof. C. Fogher, Catholic University at Piacenza, Italy). Plasmid sequences and maps are available in Supplemental file S1. The pAMFIT vector contained the *phyA* gene encoding *Aspergillus niger* phytase under the control of an α-amylase promoter, while the pAL70 vector carried the *bar* gene conferring herbicide resistance under a maize Ubiquitin promoter. Amylase promoter was chosen to specifically drive the expression in caryopsis during germination and to some extent in developing grain. Anthers from the tetraploid barley T1 regenerant HH3E/8, which had integrated both transgenes, were cultivated on MN6 medium^30^ to reduce the ploidy level. Of the 15 plants (T2 generation) obtained, only plant No. 121 maintained the 4n ploidy level and contained both transgenes. Seeds (T3 generation) from the No. 121 line were planted in a greenhouse (16/8 h day/night, 20/18°C). The ploidy level and transgene heritability were evaluated in 28 plants. Anthers from 20 plants were isolated and cultivated on MN6 medium^30^ to derive the T4 generation. Diploid plants of the T4 generation with both transgenes stably inherited were selected for further experimentation.

### Genotyping

Genomic DNA was extracted from the young leaves of plants grown in greenhouse and field conditions to detect the presence of *phyA* and *bar* transgenes in the transgenic lines.^31^ The DNA quantity was spectrophotometrically assessed and the samples were diluted at 200 ng/μL. PCR detection using a REDTaq® ReadyMix™ PCR Reaction Mix (R2523, Sigma-Aldrich) was employed to verify the presence of the transgenes. The DNA primer sequences used for *phyA* and *bar* detection are provided in Table S1. The amplified products were separated on a 1% agarose gel and visualized following ethidium bromide staining.

### Crossing of Phytase Transgenic Line with Feed Cultivar Azit

Homozygous transgenic barley lines 1015–7 and 880 over-expressing *phyA* and *bar* transgenes at the T5 generation were crossed with the feed cultivar Azit, which was chosen as the pollen donor. The plants were grown under the greenhouse conditions described previously. Immature anthers were removed from the transgenic lines using forceps. Emasculation of the female parent was performed in the range of 2–4 days in advance of anthesis. The flag leaf was carefully unrolled from the spike, the spikelets were cut off, and the anthers were removed using forceps. The spike, now free of all anthers, was isolated using a paper bag. After two to five days, depending on the development of the female organs, the pollen was applied to the spike using forceps, and the spike was isolated again. The hybrid seeds were harvested 35 to 45 days later. The F1 generation was screened for the presence of the *phyA* and *bar* transgenes. The highly polymorphic barley genetic microsatellite marker *EBmac0603* was used to confirm successful hybridization. Androgenesis was employed to stabilize the transgenes in confirmed hybrid progeny carrying the *phyA* and *bar* transgenes. The methodology was described in.^32^

### Ploidy Level

The ploidy level of transgenic plants was evaluated based on flow-cytometric determination of nuclear DNA content using flow-cytometer PARTEC II.^33^

### Copy Number Assessment

The copy numbers of the *bar* and *phyA* transgenes were assessed using qPCR as described in Bartlett et al.^34^ by iDNA Genetics (John Innes Center, Norwich). Transgene copy numbers were evaluated for both transgenes in the hybrid plants (Azit ×Golden Promise) as well as the T1 and T2 generation transgenic plants. DNA was extracted by the same method used for the hpt PCR (described above). Quantitative real-time PCR was carried out by iDNA Genetics using luciferase and CO2 (Constans-like, AF490469) gene specific primers and probes. Primers were designed to the target sequences using Applied Biosystems software Primer Express. The reactions used ABGene Absolute QPCR Rox Mix (Cat number AB1139). The luciferase gene and the CO2 gene were assayed in multiplex. The probes and primer final concentrations were 200 nM. The assay contained 5 µl of DNA solution, and was optimized for final DNA concentrations 1.25 to 10 ng/µl (6.25 to 50 ng DNA in the assay). PCRs were carried out in an Applied Biosystems ABI7900 equipped with a 384 place plate. The detectors used were FAM-TAMRA and VIC-TAMRA with Rox internal passive reference, and no 9600 emulation. The PCR cycling conditions were 50°C 2 minutes hold, 95°C 10 minutes (enzyme activation), 40 cycles of 95°C 15 seconds, 60°C 60 seconds.

### Field Trials

**Figure 1.**
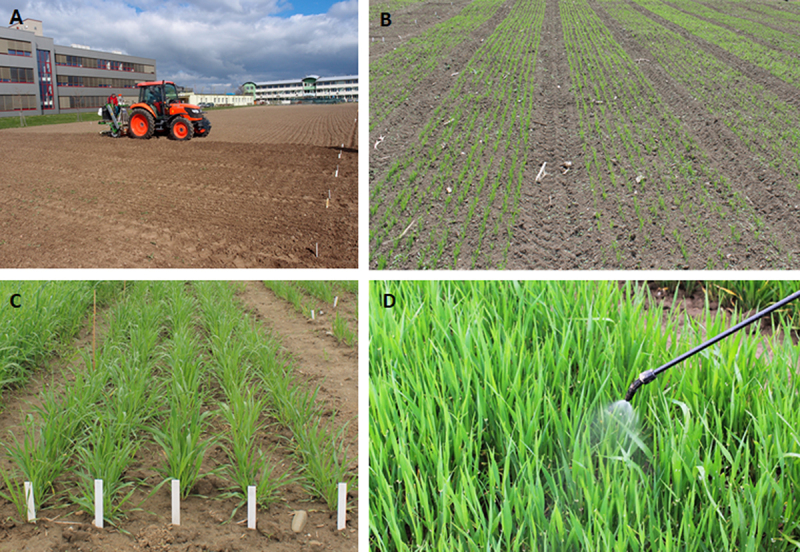
Sowing at Olomouc locality in 2015. A, Sowing of transgenic spring barley by seeder HALDRUP SB-25. B, Spring barley seedlings, barley development stage 15, 3 weeks after sowing. C, Barley development stage 30. D, Barley development stage 32, application of basta herbicide.

**Figure 2.**
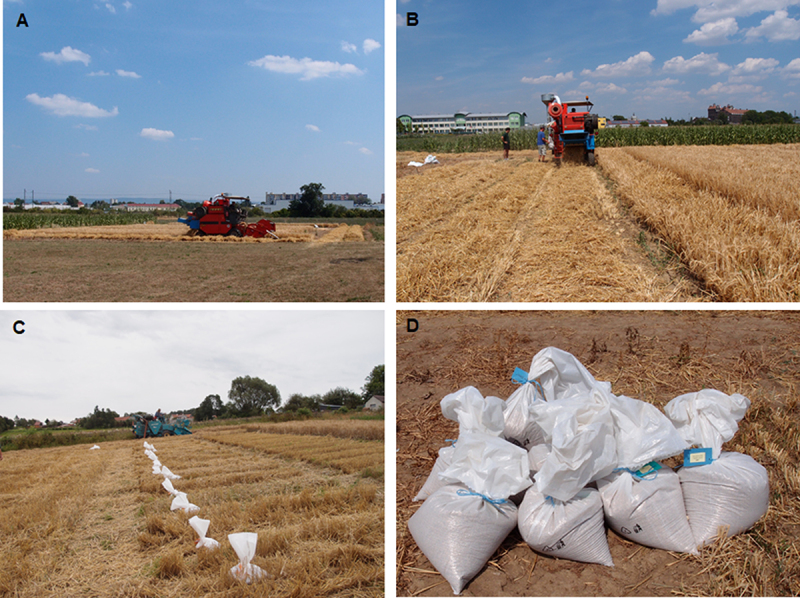
Harvest at Olomouc and Luavec locality in 2015. A, B, transgenic spring barley SCLW-GP-PHYA harvest by WINTERSTEIGER delta in Olomouc. C, transgenic spring barley SCLW-GP-PHYA harvest by HAMSTER EP 500 in Lukavec. D, harvested grains in sacks with labels.

Transgenic barley over-expressing the phytase gene *phyA* was registered as a utility model named SCLW-GP-PHYA (SYSNO ASEP number: 0369736, http://hdl.handle.net/11104/0203734). Transgenic barley was released into the environment with the permission of the Ministry of the Environment of the Czech Republic at Lukavec locality in 2011 and Olomouc locality in 2012. The field trial was conducted over five consecutive growing seasons. The two experimental locations differed in elevation, soil type, and climate. The meteorological data, including temperature and precipitation values, from the sites where the field experiments were performed were obtained from The Czech Hydrometeorological Institute (www.chmi.cz) and are shown in Table S2. The Olomouc site was a lowland area with an average elevation of 200 meters, while the Lukavec site was higher at 415 meters above sea level. The soil type was alluvial at Olomouc and brown soil at Lukavec. The experimental fields were prepared with standard fertilization (50 kg pure nutrient N/ha, 10 kg pure nutrient P/ha, 10 kg pure nutrient K/ha) and standard agricultural techniques prior to sowing. Additionally, the barley seeds were treated with a fungicide before sowing. Two transgenic barley lines, 880–4 and 1015–7, along with the control cultivar Golden Promise, were sown using a seed drill at a density of 400 grains/m^2^ (Fig. 1A). The experimental plots typically measured 1.2 × 21.0 meters, with a 0.6 m spacing between adjacent plots (Figs. 1B,C). The plots were surrounded by a protective zone planted with non-transgenic barley. Molecular analysis of the transgenic lines was conducted annually. Twenty randomly selected plants per plot were tested for the presence of *phyA* and *bar* genes before the herbicide application. The expression of the herbicide-tolerance *bar* gene was evaluated by applying a 0.5% solution of BASTA 15 (Bayer) (250 L/ha) during the tillering growth stage annually (Figure 1D). Mechanical harvesting using a combine harvester was performed (Figure 2A,B), and the grains were deposited in sacks (Figure 2C,D). The harvested grains from the transgenic lines and control were used as seeds for the following year’s sowing. Phytase activity was measured in the harvested grains of the transgenic lines and control approximately one month after harvest. Phosphate content was determined in the harvested grains in 2014 and 2015.

### Transgene Methylation Analysis

Grains from the 2013 and 2015 field trials, as well as progeny of the T3 generation cultivated in the greenhouse, were germinated on wet filter paper and then transferred to pots with paddy soil. Genomic DNA was extracted from young leaves of the transgenic plants using paramagnetic beads (SPRISelect, Backman Coulter). A method by Kanukova et al.^35^ based on phenol extraction was adopted for DNA extraction directly from grains. Genomic DNA was quantified spectrophotometrically, and diluted to 200 ng/μL. The DNA underwent bisulfite treatment using the Methylamp Universal Methylated DNA Kit (EPIGENTEK) as per the manufacturer’s protocol. A CpG island was identified within the transgene sequence, and methylation-sensitive primers covering the Amylase promoter and partial protein coding sequence were designed using the MethPrimer online tool.^36^ The Amylase promoter and protein coding sequences were then PCR-amplified using the appropriate primers on both the treated and untreated DNA samples, with the primers listed in Table S1. The amylase promoter amplicon was purified using paramagnetic beads and prepared for sequencing. During the amplification of the *phyA* protein coding sequence via PCR using methylation-sensitive primers, an unspecific amplicon was obtained. The product was gel separated and a specific amplicon extracted from the agarose gel using the QIAEX II Gel Extraction Kit, following the manufacturer’s instructions. The PCR products were sequenced by EurofinsGenomics using Sanger and Oxford Nanopore sequencing methods. Sequence alignment was subsequently performed using MegaX software.

### Phytase Activity

An indirect method was employed to quantify phytase activity. Barley grains were homogenized in a mixer mill (Retsch MM301), and 100 mg of the homogenate was transferred to a microtube. An extraction solution was added, and the samples were incubated for 40 minutes at room temperature with mixing at a frequency of 560 Hz. The samples were then centrifuged, and 100 μL of the extract was transferred to a fresh microtube with the addition of 300 μL of acetate buffer at pH 5.5. After a 5-minute pre-incubation at 37°C, 800 μL of phytate solution (Sodium phytate Sigma, Cat. no. 68388) was added, mixed, and incubated for 30 minutes at 37°C in a water bath. Subsequently, 800 μL of vanadate-molybdate reagent was added. To exclude phosphate present in the reaction before incubation from the phytase activity quantification, each sample had a variant where the phytate solution and vanadate-molybdate reagent were added to the extract without the 30-minute incubation. The difference in inorganic phosphorus, which reacts with the vanadate-molybdate reagent, before and after the 30-minute incubation in excess phytic acid, was determined. The resulting complex was assessed colorimetrically at a wavelength of 415 nm. A calibration curve was used to quantify the formed complex. The phytase activity is reported in a phytase unit (FTU), which is defined as the amount of enzyme that releases 1 μM of inorganic phosphate from phytate in 1 minute at a pH of 5.5 and a temperature of 37°C. All measurements were performed in triplicate.

### Inorganic Phosphate Determination

The colorimetric method based on the interaction between inorganic phosphorus with Chen’s reagent^37^ was used for the assessment of digestible phosphorus. The homogenized grains were mixed with 500 μL of 0.4 M hydrochloric acid in the microtube, using a 50 mg sample, and incubated overnight at 4°C. Upon centrifugation, 10 μL of extract was transferred to a 96-well plate, followed by the addition of 90 μL distilled water and 100 μL Chen’s reagent. The evolved complex was assessed colorimetrically at a wavelength of 820 nm after two hours of incubation. A calibration curve was used for quantifying the formed complex. All measurements were done in triplicate.

### Germination Experiment

The content of digestible phosphorus in the grains of the transgenic barley line SCLW-GP-PHYA 1015–7 of T5 generation during germination was characterized. In the experiment, the transgenic line and control non-transgenic barley cultivar Golden Promise were compared. Approximately 10 grams of grains were surface sterilized in 2% sodium hypochlorite and then washed three times in sterile water. Grains were sowed on wet filter paper daily over 7 days under laboratory conditions. The seeds were then dried and homogenized in a mixer mill (Retsch MM301). Phosphate content and phytase activity were determined as described above.

### Micromalting and Malt Quality Analysis

The malting of the 0.5 kg samples was carried out in a micro-malting plant (KVM Czech Republic) according to the MEBAK method^38^. The quality of malt was determined according to the methods described in MEBAK^38,39^ and EBC Analysis Committee^40^. The methods used are listed in the table (Table 1 and Table S4).

**Table 1.** Basic characteristics of malt and Wort.

Parameter	Methods				Line		
	Reference	Chapter	GP OL	GP LUK	1015–7 OL	880–4 OL	880–4 LU
Moisture content (%)	^40^	3.2	9.5	9.6	8.4	9.3	9.8
Protein content of barley* (%)	^40^	3.3.1	11.9	16.2	17.7	14.7	16.4
Total nitrogen of barley (%)	^40^	3.3.1	1.90	2.59	2.83	2.35	2.62
Water content in malt (%)	^40^	4.2	6.1	6.3	5.8	6.2	6.3
Extract of malt d.m. (%)	^40^	4.5.1	80.5	77.9	73.3	75.6	75.6
Relative extract 45°C (%)	^38^	3.1.4.11	42.6	44.4	44.7	45.1	40.2
Kolbach index (%)	^40^	4.3.1. 4.9.1	44.4	36.2	34.8	40.7	34.9
Diastatic power (WK)	^40^	4.12.2	341	338	631	458	456
Apparent final attenuation (%)	^40^	4.11.1	78.1	78.3	75.8	75.5	77.9
Friability (%)	^40^	4.15	78	44	56	67	45
β-Glucans in wort (mg/L)	^40^	4.16.2	233	639	213	256	624
Haze of wort (90°)	^40^	9.29	1.01	3.54	0.82	1.21	1.09
Haze of wort (12°)	^40^	9.29	1.30	3.45	1.25	1.99	1.30
Appearance (clarity) of wort	^39^	*R*-205.05.730	1	2	1	1	1
Wort colour (EBC)	^40^	4.7.2	3.3	4.1	4.2	4.1	3.2
α-Amylase activity (U/g)	^43^		57	64	67	67	64
GP – Golden Promise; OL – Olomouc; LU – Lukavec; 880–4 and 1015–7 - transgenic barley lines. Wort clarity: 1 – clear, 2 – weakly opalizing, 3 – opalizing, 4 – cloudy. * (factor 6.25)							

### Statistical Analysis

All statistical analyses were done using the statistical program NCSS9. Data were statistically evaluated using a two-sample student t-test and analysis of variance (ANOVA). The Bonferroni multiple comparison test was used to detect the significant differences. Error bars represent standard deviation (SD).

## Results

### Transgenic Plants and Field Trial

**Figure 3.**
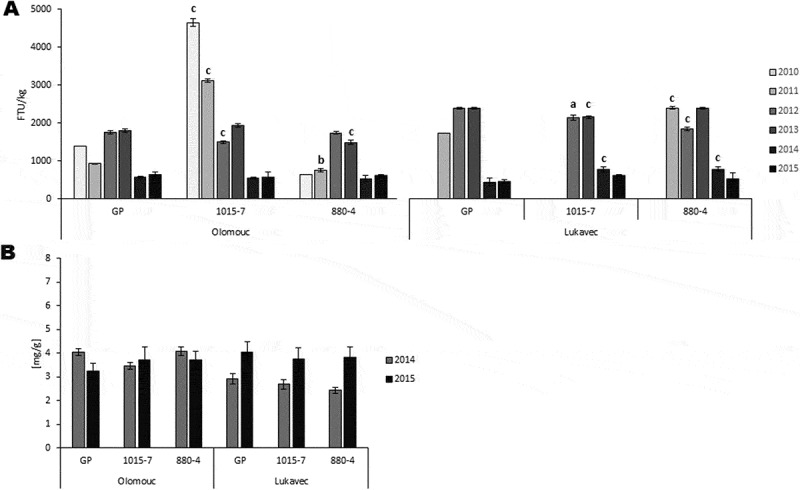
Phytase activity and phosphate content at Olomouc and Lukavec region from 2011 to 2015. A, phytase activity. B, phosphate content. Data represent means (*n* = 3 ± SD). Transgenic barley lines 880–4 and 1015–7 were compared to the control Golden promise (GP) from the same locality and harvest year. The letters represent significant differences at *p* < .05 (a), *p* < .01 (b), *p* < .001 (c) compared to GP based on a two-way ANOVA with Bonferroni post hoc test. Phytase unit (FTU) is the amount of enzyme which liberates one micromole of inorganic phosphate per minute from a sodium phytate substrate at pH 5,5 and 37°C.

Transgenic barley lines were initially cultivated under greenhouse conditions, where the presence of the *phyA* and *bar* transgenes was confirmed in all plants of the T3 generation. Approximately one month after harvest, the grains were analyzed for phytase activity. Under greenhouse conditions, the transgenic line 1015–7 showed a significantly higher phytase activity, 3.3-fold greater than the control, in both 2010 and 2011, (Figure 3A). Once a sufficient amount of grains had been produced, the field trials were initiated. The results of the field trials conducted over four and five consecutive years at the Olomouc and Lukavec locations respectively, in the Czech Republic are presented. These two sites represent distinct agricultural regions that differ in factors such as elevation and soil type.

**Figure 4.**
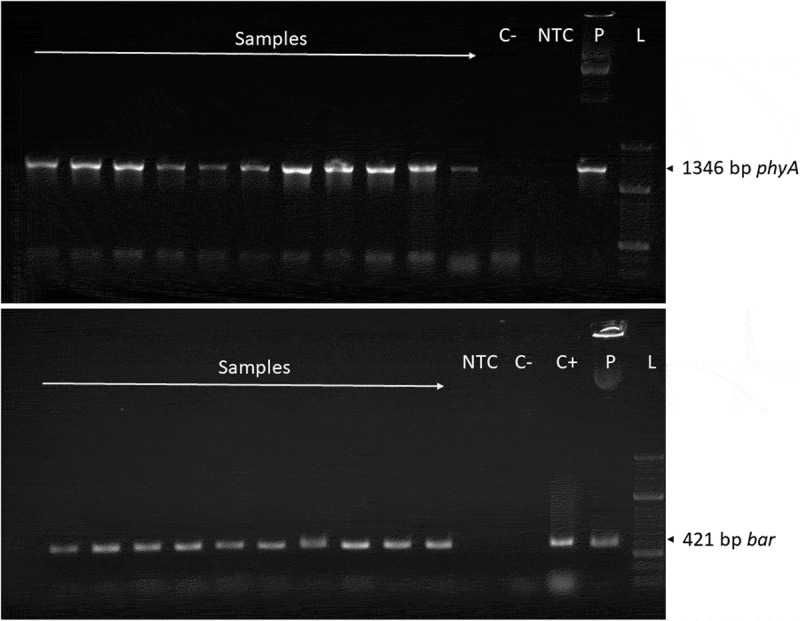
Detection of *phyA* and *bar* transgenes. molecular detection of *phyA* (1346 bp) and *bar* (421 bp) transgenes. C- – negative control, NTC – no template control, C+ - positive control, *p* – plasmid, positive control, L – ladder.

Field trials conducted at two localities in the Czech Republic from 2011 to 2015 confirmed the stable heritability of the transgenes *phyA* and *bar* up to the 10^th^ generation as shown by periodic molecular screening. The detection of DNA amplicons of the *phyA* and *bar* genes is illustrated in Figure 4. The herbicide tolerance conferred by the *bar* gene was successfully verified through the application of BASTA 15 herbicide on the developing plants. No fading of the transgenic plants was observed after the application of herbicide at any location or year, thereby functionally proving the activity of the *bar* gene product. Phytase activity and phosphate content were measured in the mature grains of the transgenic lines and the control cultivar Golden Promise, grown under field conditions one month after harvest (Figure 3A,B).

In contrast to greenhouse conditions, a remarkable decrease in phytase activity to only 85% of the wild-type level was observed for line 1015–7 in the first year of the field trial at the Olomouc locality in 2012. Conversely, line 880–4 showed a slight increase in phytase activity, though it remained at a lower level than the control. Phytase activity generally fluctuated over the course of the field trials. Focusing on the last two years, phytase activity did not differ significantly between transgenic lines and control in Olomouc. In contrast, in 2014, both transgenic lines showed a 1.7-fold higher phytase activity in Lukavec. However, in the following year, this activity reduced to 1.3-fold and 1.14-fold of the wild-type level for 1015–7 and 880–4, respectively. No significant differences in phosphate content of the mature barley grain were observed between the transgenic lines and the control from the same locality in 2014 and 2015. At the Olomouc site, phosphate levels were stable, while an increase was observed at the Lukavec locality in 2015 compared to the previous year. Overall, the phosphate content did not differ significantly between the transgenic lines and the control across locations or years. Focusing on precipitation and air temperature during vegetative growth from 2011 to 2015, we concluded that the plants experienced a warmer climate with slightly less precipitation compared to the thirty-year mean value. However, these conditions had a negligible impact on the plant phenotype, based on a comparison of transgenic and control lines. Additionally, barley yield parameters in those years were stable in the Czech Republic.

### Crossing with Feeding Cultivar

**Figure 5.**
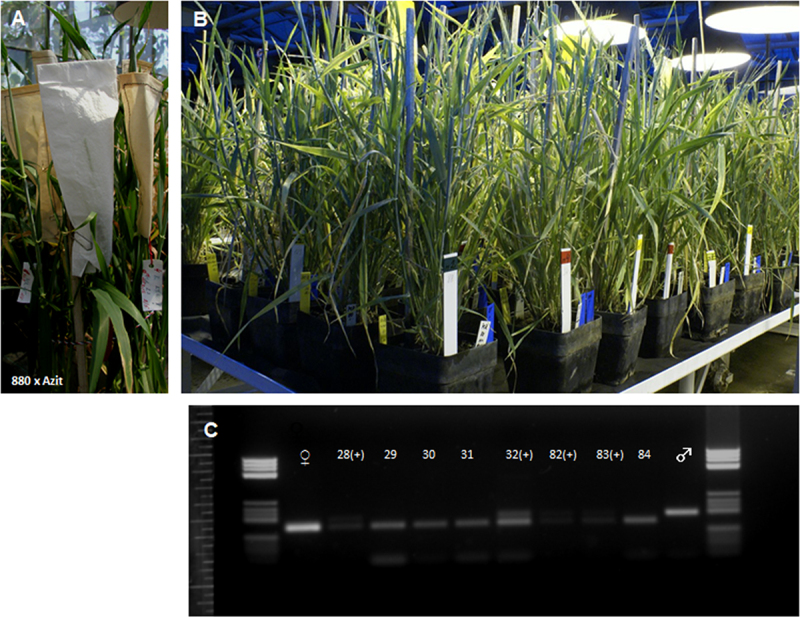
Crossing of SCLW-GP-PHYA lines with the feed spring barley variety Azit. A, isolated clusters after hybridization 880 x Azit. B, plants of F1 generation of hybrids in the greenhouse. C, verification of the presence of parental genomes in a hybrid using microsatellites *EBmac0603*, the plus symbol indicates detected hybrids.

Transgenic barley lines 1015–7 and 880, derived from the original cultivar Golden Promise, were crossed with the feeding cultivar Azit (Figure 5A,B). A total count of 141 putative hybrid plants were obtained from this cross. The majority of these plants, totally 107, exhibited the presence of both transgenes. However, plants harboring only one of the two transgenes were detected in lower numbers. The transgenic plants were analyzed using SSR markers to confirm hybridization (Figure 5C). Twenty-three plants were confirmed as true hybrids carrying both the *phyA* and *bar* transgenes.

**Figure 6.**
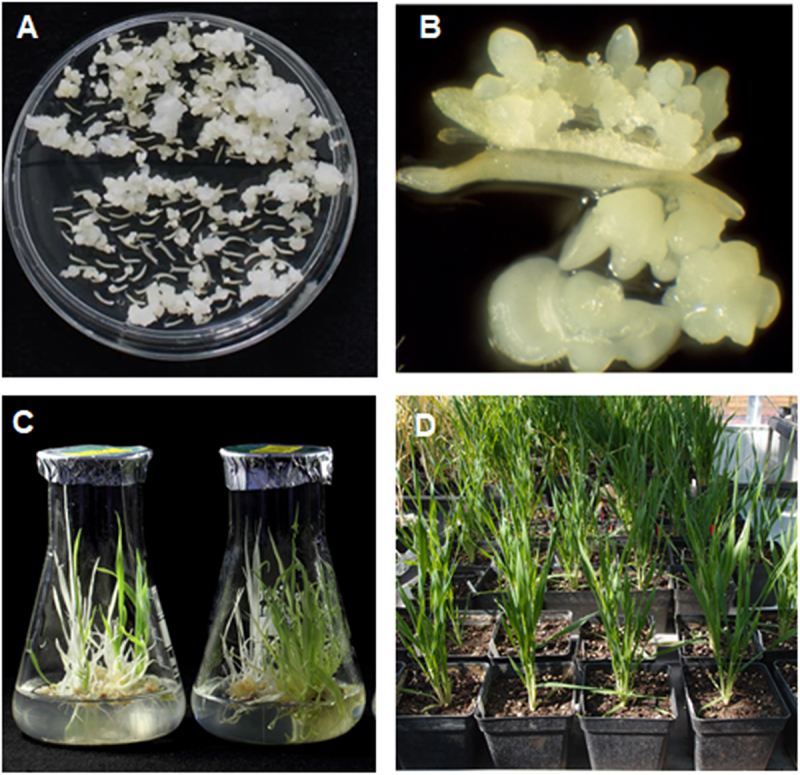
Induction and regeneration processes in barley anther culture of hybrids. A-B, formation of pollen embryos or calli on induction medium after 4–5 weeks. C, regenerating plantlets after 3 weeks on regeneration medium. D, dihaploid plants are potted and grown in the greenhouse.

**Figure 7.**
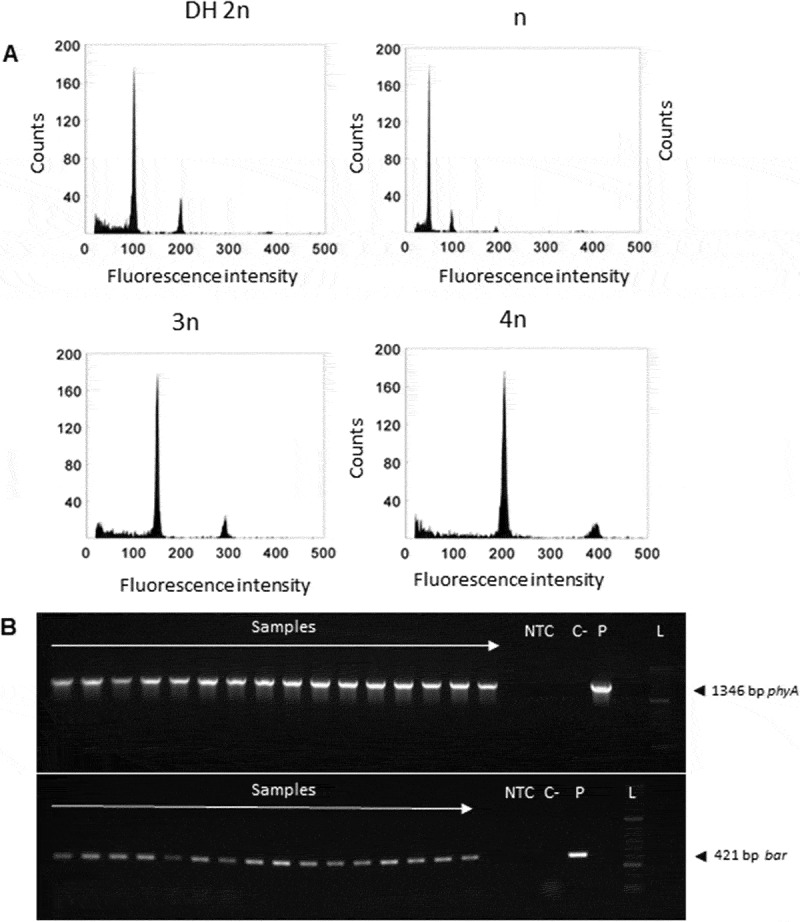
Molecular characterization of F2 generation hybrid lines. A, DNA content of nuclei isolated from leaves of regenerated plants. The position of the main peaks indicates the ploidy level. B, molecular detection of *phyA* (1346 bp) and *bar* (421 bp) transgenes. C- – negative control, NTC – no template control, *p* – plasmid, positive control, L – ladder.

**Figure 8.**
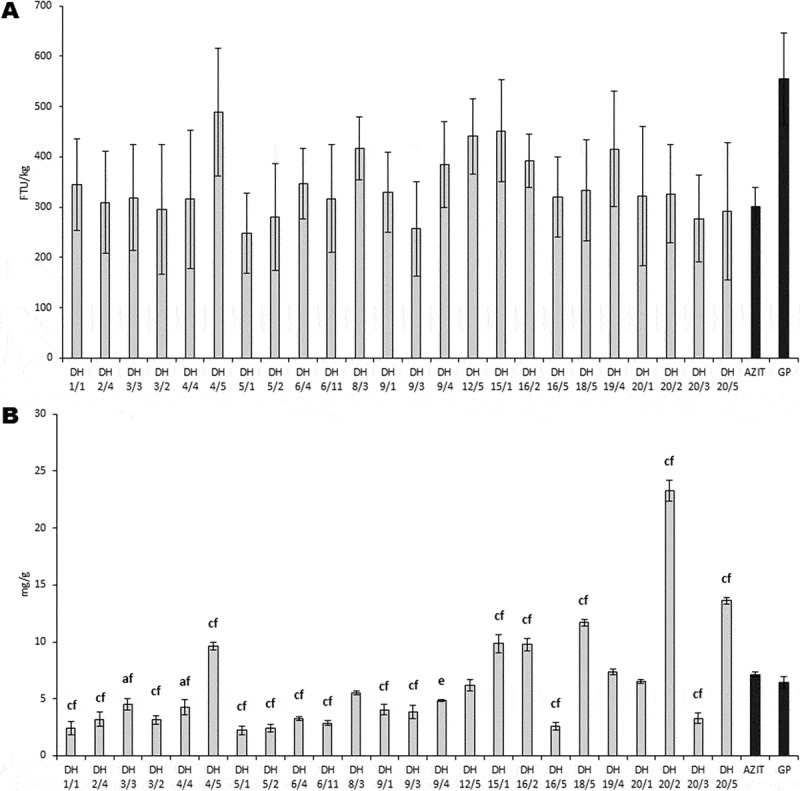
Phytase activity and phosphate content in the grains of hybrid lines. A, phytase activity. B, phosphate content. Data represent means (*n* = 3 ± SD). Transgenic hybrid barley dihaploid (DH) lines were compared to the parental Golden promise (GP) and Azit. The letters represent significant differences at *p* < .05 (a, d), *p* < .01 (b, e), *p* < .001 (c, f) compared to the control GP or Azit, respectively, based on the one-way ANOVA with Bonferroni post hoc test. Phytase unit (FTU) is the amount of enzyme which liberates one micromole of inorganic phosphate per minute from a sodium phytate substrate at pH 5,5 and 37°C.

Ten of the transgenic hybrid plants from the F2 generation were subsequently stabilized through androgenesis (Figure 6). A total of 4,834 anthers were excised and subjected to in vitro culture over several explanting cycles. From these cultures, fifty-four green and 148 albino plants were regenerated and their ploidy was evaluated (Figure 7A), and also screened for the presence of the *phyA* and *bar* transgenes (Figure 7B). The majority of plants, forty-two, exhibited a natural ploidy number of 2n, six plants had a ploidy number of n, only two plants showed 3n, and four plants showed 4n. Phytase activity and phosphate content in mature grains of homozygous transgenic doubled-haploid hybrid plants grown in the greenhouse were evaluated (Figure 8). A total of 24 doubled-haploid hybrid lines were evaluated. Phytase activity in the Azit cultivar was naturally lower than in Golden Promise, likely due to the genetic background of Azit. However, some hybrid lines exhibited improved phytase activity when compared to Azit. Phosphate content exhibited substantial variability among the studied samples, with a low positive correlation observed between phosphate content and phytase activity. Analysis of transgene copy number in selected hybrid progeny lines confirmed the presence of two copies of the *bar* and *phyA* transgenes (Table S3). The phenotype of doubled-haploid lines with confirmed presence of two transgenes varied in combinations of phenotypic traits from both parents.

### Phytase Activity and Phosphate Content Analysis During Germination

**Figure 9.**
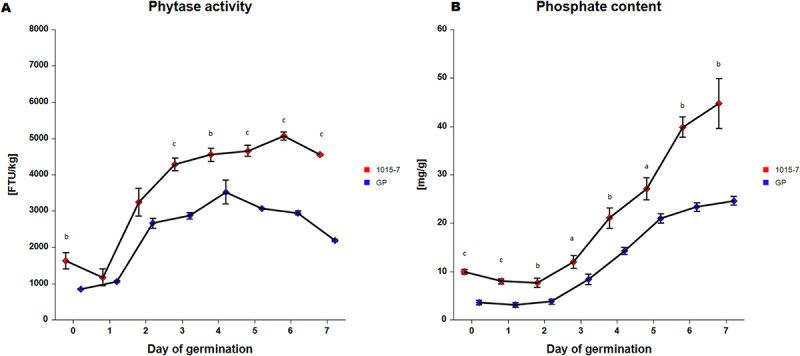
Phytase activity and phosphate content during germination. A, phytase activity. B, phosphate content. Data represent means (*n* = 3 ± SD). The letters represent significant differences at *p* < .05 (a), *p* < .01 (b), *p* < .001 (c) compared to the control Golden promise (GP) based on the two-sample t-test for equal variances. Phytase unit (FTU) is the amount of enzyme which liberates one micromole of inorganic phosphate per minute from a sodium phytate substrate at pH 5,5 and 37°C.

A germination experiment was conducted to determine the phytase activity and phosphate content in grains of transgenic barley and the control. Germination percentage was 98% in both. Phytase activity fluctuated remarkably over the seven days of germination, as shown in Figure 9A. In the transgenic barley, the slight initial decline in phytase activity on day 1 was followed by a sharp rise, peaking on day 6 at 5070 ± 90 FTU/kg. In comparison, the phytase activity of the control exhibited a similar sharp rise, reaching its maximum on day 4 at 3530 ± 270 FTU/kg. The phosphate content in the mature grain (day 0) of the transgenic line was approximately 3 times higher than the content in the control. Interestingly, the phosphate content of the transgenic barley declined for the first two days of germination, as depicted in Figure 9B. In contrast, the control line only experienced a decline in phosphate content on the first day. Thereafter, a significant increase in phosphate content was detected in both the transgenic and control lines.

### Epigenetic Analysis of the phyA Gene

**Figure 10.**
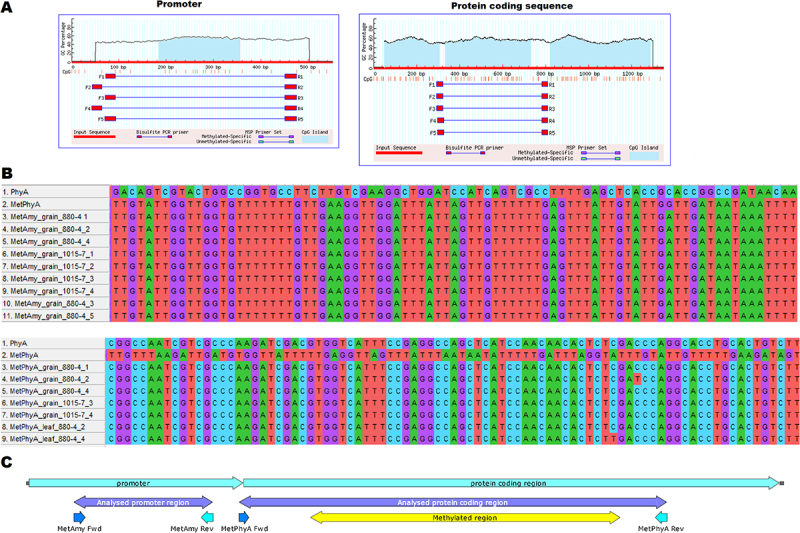
Analysis of phyA gene methylation in transgenic barley. A, detection of CpG islands in the promoter and protein coding sequence. B, multiple sequence alignment of bisulfite-treated DNA samples was performed in MEGAX with default parameters. The upper alignment shows the promoter region, and the lower alignment shows the protein-coding region. PhyA represents the original DNA sequence, while MetPhyA represents the artificially prepared fully converted DNA sequence. C, a scheme of the phyA gene with the amylase promoter was prepared in SnapGene viewer. The regions analysed for methylation are highlighted in purple, and the methylated region is highlighted in yellow.

The MethPrimer software was used to identify CpG islands in the promoter and protein coding sequence of the *phyA* gene (Figure 10A). Genomic DNA was extracted from the young leaves and grains of the barley line 1015–7 and 880–4 harvested in 2013 and 2015. The primers designed for amplification of the CpG island sequences were utilized to amplify the target regions. To characterize the methylation pattern in the *phyA* transgene sequence, sequence alignment was performed on transgenic barley lines from various generations. Comparison between treated and untreated DNA sequences revealed no methylation in the promoter of transgenic lines from any analyzed generation (Figure 10B). Conversely, a clear methylation pattern within the protein coding sequence was detected in all DNA samples extracted from grains and leaves. The results indicated that transgene methylation covered 54% of the protein coding sequence (Figure 10C).

### Malt Quality

Refer to Table 1 for main malt characteristics and Table S4 for additional results of the malt analysis of the studied samples. The control sample of the Golden Promise GP OL variety had a normal protein content (11.9%) in unmalted grain, and the malting quality of the GP OL sample approached current malting quality requirements.^41,42^ The control sample GP LUK and samples of transgenic barley lines (1015–7 OL, 880–4 OL, 880–4 LU) had very high protein content in unmalted barley grain, which negatively affected their malting quality. The protein content in barley grain is easily influenced by external factors (weather conditions, fertilization, etc.).

Samples of transgenic barley lines (1015–7 OL, 880–4 OL, 880–4 LU) had very high protein content (14.7–17.7%) in unmalted grain. The extract content (73.3–75.6%) was very low, while the diastatic power (456–631 WK) was at an optimal level. The intensity of proteolytic modification was very low in samples 1015–7 OL and 880–4 LU (Kolbach index 34.8, 34.9%), but sample 880–4 OL reached acceptable values (Kolbach index 40.7%). The degradation of cell walls (friability 45–67%) was very slow, which corresponded to the very high β-glucan content of malt wort (213–624 mg/l). The wort of these samples did not have a satisfactory composition, which was reflected in the low apparent final attenuation (45–67%). The samples provided mostly clear wort, and the color of the wort corresponded to the color of pale malt.

## Discussion

### Field Trials

Field trials represent a basic yet powerful means of evaluating crops under environmental conditions, including biotic and abiotic stresses. These trials have a higher predictive value than greenhouse experiments. For instance, field experiments with heterologous phytase-expressing corn were conducted in China.^44,45^ While these studies focused on the environmental aspects, they provided evidence that transgenic plants had no negative effect on the arthropods. An experiment with cisgenic barley over-expressing phytase released into the environment in Denmark lacked long-term evaluation of phytase activity under the field conditions.^46^ Here, two lines of transgenic spring barley SCLW-GP-PHYA were evaluated in field trials at two locations, focusing on the stability of phytase activity and herbicide resistance over several growing seasons. Furthermore, practical applications of transgenic barley were demonstrated through hybridization with a feeding cultivar and an assessment of brewing characteristics. Mature barley grains naturally exhibit phytase activity.^47^ Additionally, the over-expression of phytase significantly increases these levels. Our results demonstrate that phytase activity in line 1015–7 was significantly higher than the control under greenhouse conditions. In contrast, field trial with natural environmental conditions resulted in a gradual unification of phytase activity across the studied transgenic lines and the control. Phosphate content remained more stable among the tested genotypes. Surprisingly, our data indicates a negative correlation between phytase activity and phosphate content in mature grains from field trials. The parameters were rather influenced by locality and year. Similarly, a lack of correlation between phytase activity and phytate content in mature grains was noticed in Tibetan wild accessions.^48^

While the activity of the *phyA* gene appeared to be suppressed more rapidly under field conditions, the *bar* gene products providing herbicide resistance against BASTA 15 did not seem to be affected. Consequently, the *bar* gene activity exhibited more stable expression over the years in the field trial. The annual BASTA 15 treatment verified the activity of the *bar* gene. Analogously, stable expression of the *bar* gene over generations was reported by Bregitzer and Brown.^49^

### Hybridization

The concept of transgenic barley-producing fungal phytase was to enhance phosphate digestibility in barley-fed non-ruminants. Since Golden Promise is not a modern feeding variety, homozygous transgenic barley from the field trials was crossed with the feeding cultivar Azit to transfer the transgene into a more contemporary cultivar. This demonstrates that transgenic barley can be readily crossed with a feeding cultivar. Furthermore, the use of doubled-haploid technology to stabilize the hybrid progeny proved to be an efficient and straightforward time-saving approach, as opposed to the F2 generation screening method employed by Choi et al..^50^ We obtained several hybrid lines carrying both transgenes, and some of these lines exhibited increased phytase activity and improved phosphate content compared to the feeding cultivar Azit.

### Germination

In germination, a clear positive correlation was observed between phosphate content and phytase activity.^51–55^ In our study, the phytase transgene was placed under the control of an amylase seed-specific promoter. Amylases are *de novo* synthesized during germination, and their activity rises, reaching a peak on the fourth day of germination.^56^ Characterization of phytase activity and phosphate content during germination revealed improved parameters in the transgenic line. Phytase activity in the control line increased from day 1, reaching a peak on day 4, after which it gradually decreased. A similar pattern of phytase activity was observed in non-transgenic barley grains.^53,54,57^ However, the time point when phytase activity reaches its peak can vary depending on the germinating conditions. For example, Centeno et al.^51^ observed gradually increasing phytase activity that reached its maximum on the fifth day of germination. Likewise, Sung et al.^54^ reported that the day when maximal grain phytase activity is attained during germination is temperature dependent. Compared to the control, the transgenic line exhibited increasing activity until day 6. The two-day delay in reaching peak phytase activity in the transgenic barley line was presumably caused by the prolonged expression of the heterologous phytase that was driven by the amylase promoter. Since amylase expression continues until day 6,^56^ it can be expected that the duration of heterologous phytase expression occurred during this same period. The slight decrease in phosphate content on day one could have been caused by the utilization of phosphate already present during the initial phase of germination. Similarly, the decrease in phytase activity in the transgenic line suggests that phytase enzymes, presumably phyA present in mature grain, were hydrolyzed because phytase action was not necessary due to the relative abundance of phosphate. Once the phosphate level decreased and protein biosynthesis was activated, increased phytase activity resulted in a higher phosphate content.

### DNA Methylation Analysis

DNA methylation is an epigenetic mechanism that induces dynamic changes in gene expression, primarily down-regulation. Transgene silencing in plants has been reported to occur at either the transcriptional or post-transcriptional level.^58^ Meng et al^59^ reported that environmental stresses should not impact transgene stability. Fan et al.^60^ summarized works discussing the effect of drought stress on methylation patterns emphasizing the scarcity of data from field trials. Our findings show that the *phyA* gene has become silenced, while the *bar* transgene was active over all generations in both greenhouse and field conditions. Stable expression of a transgene in barley was documented up to the T5 generation when a hordein seed-specific promoter was used.^61^ Unfortunately, this was the case for a reporter gene that presumably did not substantially alter cell metabolism. The significant reduction in phytase activity observed during our field trials under real environmental conditions suggests that the heterologous expression of fungal phytase phyA was likely subject to epigenetic down-regulation. Interestingly, we found strong methylation in the gene body region, but not in the promoter or 5’UTR region. Gene body methylation is an evolutionarily developed phenomenon reported to be associated with expression levels; however, studies on its function are inconsistent.^62^ The advantages of using long-term field trials allowed us to study epigenetic changes under realistic and variable environmental conditions.

### Malting

In the past, low phytic acid barley lines have been tested for malting parameters. However, these lines, which provided malt with higher mineral content, showed non-significant effects on fermentation efficiency.^63^ We explored the effect of heterologous phytase expression in transgenic barley on malting parameters. Our interest was to evaluate whether phytase, through increased phosphate and microelement availability, could affect malting and, consequently, biochemical processes in brewing. Qiu and Lu^64^ reported that addition of phytase during steeping increased activity of key malting enzymes such as amylases, proteinases, glucanase and xylanase thus reducing malting time. Our results showed that the control samples of the Golden Promise variety (GP OL, GP LUK) provided malt quality corresponding to the time when this variety was bred.^65,66^ Modern cultivars exhibit improved malting characteristics compared to the Golden Promise variety, which was registered in 1966. The transgenic lines assessed did not meet current quality standards, particularly regarding the concentration of nitrogenous compounds, which is considered the most important parameter for malting barley. The high protein content in unmalted barley grain affected most of the important malting traits. For instance, the malt extract, which is a crucial economic trait of malt, was low in all samples of transgenic barley lines and the control sample GP LUK. Based on the results we can conclude that locality had a great impact on malting quality. In contrast, the control sample of the Golden Promise GP OL variety closely met the current malting quality standards for barley varieties.^41,42^ The protein content in barley grain is easily influenced by external factors (weather conditions, fertilization, etc.). Indeed, the location has a major impact on proteome of barley grains and consequently affects malting quality parameters.^67^

## Conclusions

The phytase activity in food and feedstuffs is an important nutritional parameter. This study extensively characterized the transgenic barley SCLW-GP-PHYA over-expressing microbial phytase. Field trials were conducted over five years at two locations in the Czech Republic. Phytase over-expression significantly improved phosphate levels during germination. The altered phytase activity in the mature grains of the transgenic lines was accompanied by an increased protein content, which subsequently negatively affected the quality of the produced malt. Notably, the high mature grain phytase activity phenotype was lost after six generations, reverting to wild-type levels. Significant reductions in phytase activity were observed during the initial year of field trials, indicating a critical timeframe for evaluating the desired phenotype of the genetically modified plants. In contrast, the *bar* transgene, whose product did not directly affect plant metabolism, did not appear to be down-regulated. These findings demonstrate that strong greenhouse phenotypes can vary dramatically under field conditions, especially when the transgenic trait disrupts the plant’s natural homeostasis.

### Summary Points

- The increased phytase activity in transgenic lines exhibited a gradual reduction during field trials, while the selection gene’s activity remained stable.
- Phytase over-expression had no significant positive effect on the malting quality of transgenic barley.
- Androgenesis was successfully applied to obtain homozygous lines after transformation and crossing into the elite feeding spring barley variety.

## Supplementary Material

rev Table S3 Copy Number.jpg

rev Table S2 Precipitates and temperature.xlsx

rev Table S1 Primer sequences v2.docx

rev Table S4 Microbrewing Other characteristics.docx

## Data Availability

The data have been deposited in the public Zenodo repository. DOI number: 10.5281/zenodo.16794237.
