# Supplementary material for: Transgenic barley over-expressing Aspergillus niger phytase phyA in field trials
Source: GM Crops Food. 2025 Sep 15;16(1):607–25. doi: 10.1080/21645698.2025.2559488 (PMC12439572; doi:10.1080/21645698.2025.2559488)
Supplement: rev Table S3 Copy Number.jpg [file KGMC_A_2559488_SM7107.jpg]

Sample	CopiesBar_ByDeltaCt	CopiesBar_ByRegression	Copies_Phyt_byDeltaCt	Phyt_ByN_integer	Corr(regr)_Integer
DH 1-4	2	2	2	3	3
DH 15-1	2	2	2	2	2
DH 15-4	2	2	2	3	3
DHJ 1-1	2	2	1 to 2	2	2
Golden Promise_2\1_1A	0	0	0	0	0
Golden Promise_2\3_1B	0	0	0	0	0
Golden Promise_2\4_1C	0	0	0	0	0
Golden Promise_2\5_1D	0	0	0	0	0
Golden Promise_2\6_1E	0	0	0	0	0
Golden Promise_2\7_1F	0	0	0	0	0
GP UK 99	0	0	0	0	0
T1HH1A_1\1_7F	2	2	4	4	4
T1HH1A_2\1_7A	2	2	4	4	4
T1HH1A_2\5_7B	2	2	4	4	4
T1HH1A_2\7_7C	2	2	4	4	4
T1HH1A_2\8_7D	2	2	4	4	4
T1HH1A_2\9_7E	2	2	4	4	4
T1HH2A_1\10_3E	2	2	31	28	17
T1HH2A_1\3_3C	5	5	62	71	33
T1HH2A_1\7_3D	4	4	62	51	27
T1HH2A_2\10_3F	2	2	31	30	18
T1HH2A_2\3_3G	3	3	62	55	27
T1HH2A_2\5_3H	5 to 6	5	62	60	28
T1HH2I_1\4_4C	2	2	31	30	18
T1HH2I_1\7_4D	2	2	31	29	17
T1HH2I_1\8_4E	6	5	62	78	35
T1HH2I_1\9_4F	4	4	62	49	25
T1HH2I_2\2_4A	6	5	62	65	30
T1HH2I_2\3_4B	2	2	31	27	16
T1HH3A_1\1_1G	0	0	0	0	0
T1HH3A_1\3_1H	0	0	0	0	0
T1HH3A_1\6_2A	1	1	1	1	1
T1HH3A_1\7_2B	0	0	0	0	0
T1HH3A_2\1_2C	0	0	0	0	0
T1HH3A_2\2_2D	1	1	1	1	1
T1HH3E_1\1_2E	2	2	2	2	2
T1HH3E_1\2_2F	2	2	2	2	2
T1HH3E_1\3_2G	1	1	1	1	1
T1HH3E_1\4_2H	1	1	1	1	1
T1HH3E_1\6_3A	1	1	1	1	1
T1HH3E_1\9_3B	1	1	1	1	1
T2HH2I[2]_1\1_5E	2	2	31	33	20
T2HH2I[2]_1\3_5F	2	2	31	34	20
T2HH2I[2]_1\6_5H	2	2	31	28	17
T2HH2I[2]_1\7_5G	2	2	31	32	19
T2HH2I[2]_1\8_6A	2	2	31	28	17
T2HH2I[2]_1\9_6B	2	2	31	27	16
T2HH3A[3]_1\10_5D	2	2	2	2	3
T2HH3A[3]_1\9_5C	2	2	2	2	3
T2HH3A[3]_2\1_4G	2	2	2	2	2
T2HH3A[3]_2\2_4H	2	2	2	2	2
T2HH3A[3]_2\3_5A	2	2	2	2	2
T2HH3A[3]_2\5_5B	2	2	2	2	2
T2HH3E[8]_2\10_6H	1	1	1	1	1
T2HH3E[8]_2\3_6C	0	0	0	0	0
T2HH3E[8]_2\4_6D	2	2	2	2	2
T2HH3E[8]_2\5_6E	1	1	1	1	1
T2HH3E[8]_2\7_6F	1	1	1	1	1
T2HH3E[8]_2\8_6G	1	1	1	1	1

This analysis was carried out in the period 19 -28 August 2014, JIC
