# Supplementary material for: Transgenic barley over-expressing Aspergillus niger phytase phyA in field trials
Source: GM Crops Food. 2025 Sep 15;16(1):607–25. doi: 10.1080/21645698.2025.2559488 (PMC12439572; doi:10.1080/21645698.2025.2559488)
Supplement: rev Table S1 Primer sequences v2.docx [file KGMC_A_2559488_SM7105.docx]

Table S1 Primer sequences

| Gene | Sequence | Product (bp) |
| --- | --- | --- |
| phyA | GGCAGTCCCCGCCTCGAGAAA | 1346 |
|  | AAACACTCCGCCCAATCACCC |  |
| bar | GGTCTGCACCATCGTCAACC | 421 |
|  | GTCATGCCAGTTCCCGTGCT |  |
| EBmac0603 | ACCGAAACTAAATGAACTACTTCG |  |
|  | TGCAAACTGTGCTATTAAGGG |  |
| Amylase promoter | TCGAGAAACGCACCTCATCC | 662 |
|  | CAGAGAGAAGAACGGTGCGT |  |
| Met Amylase promoter | GGTGTAATAGGATAATTGATAGGAATTGTT | 350 |
|  | CCATAATCTTAAACTCTAAACTCTAACTAA |  |
| Met PhyA cds | GGGTAATTTAGAGGATTGGTAGTTTT | 1076 |
|  | CCTAAAACAAAAAAAATAAAAATATAACC |  |
