# Supplementary material for: Transgenic barley over-expressing Aspergillus niger phytase phyA in field trials
Source: GM Crops Food. 2025 Sep 15;16(1):607–25. doi: 10.1080/21645698.2025.2559488 (PMC12439572; doi:10.1080/21645698.2025.2559488)
Supplement: rev Table S4 Microbrewing Other characteristics.docx [file KGMC_A_2559488_SM7104.docx]

| Parameter | Methods | |  |  | Line |  |  |  |  |  |  |  |  |  |  |
| --- | --- | --- | --- | --- | --- | --- | --- | --- | --- | --- | --- | --- | --- | --- | --- |
|  |  |  | GP OL | GP LUK | 1015-7 OL | 880-4 OL | 880-4 LU |  |  |  |  |  |  |  |  |
| \| Protein in malt* (%) \| EBC 2010 \| 4.3.1 \| 11.6 \| 15.9 \| 17.3 \| 14.4 \| 16.1 \| \| --- \| --- \| --- \| --- \| --- \| --- \| --- \| --- \| \| Total nitrogen in malt (%) \| EBC 2010 \| 4.3.1 \| 1.86 \| 2.54 \| 2.77 \| 2.31 \| 2.58 \| \| Soluble nitrogen in wort (mg/L) \| EBC 2010 \| 4.9.1 \| 824 \| 922 \| 965 \| 941 \| 899 \| \| Wholly unmodified grains (%) \| EBC 2010 \| 4.15 \| 0.2 \| 10.6 \| 0.6 \| 0.3 \| 3.6 \| \| Homogeneity by friabilimeter (%) \| Baxter. O’Farrell \| (1983) \| 95.5 \| 66.2 \| 94.7 \| 97.0 \| 82.6 \| \| Partly unmodified grains (%) \| EBC 2010 \| 4.15 \| 4.5 \| 33.8 \| 5.3 \| 3.0 \| 17.4 \| \| pH of Wort \| MEBAK 2018 \| R-205.06.040 \| 5.89 \| 5.96 \| 5.84 \| 5.84 \| 5.87 \| \| Wort viscosity (mPas) \| EBC 2010 \| 4.8 \| 1.48 \| 1.64 \| 1.52 \| 1.51 \| 1.67 \| \|  \|  \|  \|  \|  \|  \|  \|  \| \| Wort colour after boiling (EBC) \| EBC 2010 \| 4.19 \| 6.8 \| 7.1 \| 7.2 \| 7.7 \| 6.2 \| \| Saccharification time (min) \| EBC 2010 \| 4.5.1 \| 10 \| 10 \| 10 \| 10 \| 10 \| \| Ferulic acid content in wort (mg/L) \| Běláková et al. \| (2010) \| 2.02 \| 0.94 \| 3.86 \| 2.96 \| 1.57 \| \| Oxalates in wort (mg/100 g) \| Havlová. Šusta \| (1997) \| 15.3 \| 20.2 \| 15.7 \| 14.6 \| 17.8 \| | | | | | | | | |  |  |  |  |  |  |  |
| GP – Golden Promise; OL – Olomouc; LU – Lukavec; 880-4 and 1015-7 - transgenic barley lines. * (factor 6.25) | | | | | | | |  |  |  |  |  |  |  |  |
